# Plasma acylcarnitine and diabetic retinopathy: A study from Eastern China

**DOI:** 10.3389/fendo.2022.977428

**Published:** 2022-10-27

**Authors:** Dongzhen Jin, Shuzhen Zhao, Huihui Li, Zhezheng Xia, Mingzhu Che, Ruogu Huang, Mengyuan Lai, Yanan Wang, Zejie Zhang, Hui Wang, Jingjing Zuo, Chao Zheng, Guangyun Mao

**Affiliations:** ^1^ Division of Epidemiology and Health Statistics, Department of Preventive Medicine, School of Public Health and Management, Wenzhou Medical University, Wenzhou, China; ^2^ Eye Hospital and School of Ophthalmology and Optometry, Wenzhou Medical University, Wenzhou, China; ^3^ National Clinical Research Center for Ocular Diseases, Wenzhou Medical University, Wenzhou, China; ^4^ The Second Affiliated Hospital of Zhejiang University School of Medicine, Hangzhou, China

**Keywords:** plasma acylcarnitines, type 2 diabetes, diabetic retinopathy, propensity score matching, Acylcarnitine 8:0

## Abstract

**Background and purpose:**

Acylcarnitines (ACars) are important for insulin resistance and type 2 diabetes (T2D). However, their roles in diabetic retinopathy (DR) remain controversial. In this study, we aimed to investigate the association of ACars with DR and their values in DR detection.

**Methods:**

This was a two-center case-control study based on the propensity score matching approach between August 2017 to June 2018 in Eastern China. Multivariable logistic regression models were applied to estimate the association of plasma ACars with DR. Differential ACars were screened by models of least absolute shrinkage and selection operator, elastic net, and weighted quantile sum regression, and their roles in DR identification were further evaluated by the area under the receiver operating curve (AUC).

**Results:**

Eight of twenty plasma ACars (8:0, 12:0, 12:1, 14:1, 16:2, 18:0, 18:2 and 18:3) were associated with DR, while only ACar 8:0 was selected by three variable selection methods. As compared to those with the 1^st^ tertile of ACar 8:0, the adjusted odds ratio (OR) and 95% confidence interval (CI) of DR were 0.22 (0.08, 0.59) and 0.12 (0.04, 0.36) for subjects in the 2^nd^ and 3^rd^ tertiles, respectively (P for trend < 0.001). Consistent associations were also observed in both restricted cubic spline regression models and subgroup analyses. AUC (95% CI) were 0.74 (0.66, 0.82) for ACar 8:0 alone and 0.77 (0.70, 0.85) for ACar 8:0 combined with covariates.

**Conclusions:**

Our findings suggest higher ACar 8:0 is significantly associated with a decreased risk of DR, which provides a unique window for early identification of DR.

## Introduction

Diabetes mellitus (DM) is spiraling out of control and leads to a variety of important diseases (1). The number of diabetic retinopathy (DR), a chronic eye disease caused by DM, has also increased rapidly over the past few decades and is expected to rise from 93 million in 2012 to 224 million in 2040 (2). Due to the insidious onset of DR, almost all patients are diagnosed at a moderate or advanced stage (3), which not only leads to a decrease in clinical efficacy but also results in a huge waste of limited healthcare budgets. Therefore, it is critical to identify potential biomarkers to prevent and screen for DR.

It is reported that strict glycemic control cannot completely prevent the development from DM to DR (4), while disorders of lipid metabolism may play a key role in the pathogenesis of DR (5). A recent study revealed that carnitine metabolites were altered in patients with DR compared to those without DR (6), which provides a unique window for early identification of DR through novel lipid biomarkers. Acylcarnitine (ACar) is one of the acetyl derivatives of L-carnitine which plays a key role in the β-oxidation of long-chain fatty acids through the inner mitochondrial membrane (7, 8). Therefore, the accumulation of ACar is regarded as a response to the dysregulation of fatty acid oxidation (9). Available evidence shows that the level of specific ACars is different in patients with obesity, insulin resistance, metabolic syndrome, and diabetes compared to healthy participants (10–13). However, studies on the relationship between ACar and DR are scarce. An untargeted metabolomics study *via* high-resolution mass spectrometry found that plasma levels of deoxynivalenol in patients with diabetic retinopathy were higher than in diabetic controls (6). Another more intensive study from northern China found that multiple ACars (C2, C14DC, C16, C18:1OH, C18:1) were negatively associated with risk of retinopathy in patients with type 2 diabetes (14). However, the evidence based on population remains limited and the relationship of ACars with DR is controversial. In addition, an important question on whether ACars can serve as appropriate biomarkers for DR identification is not yet fully answered, either.

In this study, we aim to clarify the relationship between DR and plasma ACars, as well as to assess the role of plasma ACars in distinguishing DR from type 2 diabetes mellitus. These may be helpful to further elucidate the pathogenesis of DR and provide new insights into the possibility of new treatment development.

## Materials and methods

### Research subjects

This was a two-center case-control study, which is a propensity score matching (PSM) based lipidomics study conducted in China between August 2017 and June 2018. Detailed information on this study design and participants can be found in our previous works (15–17). In brief, a total of 195 patients with type 2 diabetes (T2D) patients (83 with DR and 112 without DR) aged over 35 years and free from type 1 diabetes, cardiovascular diseases, cerebrovascular diseases, hyperkalemia, cancers, infectious diseases, or other chronic systemic diseases were recruited from two affiliated hospitals of Wenzhou Medical University and Anhui Medical University. To adjust for potential effects of possible confounders and improve the comparability of the results to some extent, the PSM approach was applied, in which T2D patients with DR (case) and those without DR (control) were matched at a ratio of 1:1 by age, sex, body mass index (BMI), blood pressure (BP) and glycated hemoglobin A1c (HbA1c) (**Supplementary Figure 1**). Finally, a total of 69 pairs of cases and controls were included in this study. The cases included 58 with non-proliferative diabetic retinopathy (NPDR: 6 mild, 27 moderate, and 25 severe) and 11 with proliferative diabetic retinopathy (PDR).

### Definition of DM and DR

T2D was defined as self-reported doctor diagnosis of diabetes, use of insulin or oral hypoglycemic medication, or according to the standard criteria recommended by the World Health Organization (WHO): venous plasma glucose concentration ≥ 11.1 mmol/L (1 mmol/L = 18 g/L) or fasting plasma glucose concentration > 7.0 mmol/L or the 2h glucose concentration ≥ 11.1 mmol/L in oral glucose tolerance test (OGTT) (18). The determination of DR was independently assessed by two experienced ophthalmologists strictly following the clinical diagnostic and image screening guidelines (19). The stages of DR were further graded as mild non-proliferative DR (NPDR), moderate NPDR, severe NPDR, and proliferative DR (PDR) by the same ophthalmologists. The details on the DR diagnosis can be found in the Supplementary Materials of this manuscript.

### Demographic and clinical variables

Demographic variables including age, gender, duration of diabetes, smoking habits, alcohol consumption, occupation, and education were obtained by using a standardized questionnaire. This procedure was carried out by two systematically trained researchers strictly following the standard operation procedures (SOP) and quality control protocols specially prepared for this study. Besides, all participants also received anthropometric measurements such as weight, height, and blood pressure. Body mass index (BMI) was calculated using a formula as BMI = weight (kg)/height (m)^2^. After at least 8 hours of fasting, the blood of each participant was collected, separated into serum and plasma, and stored at -86°C strictly following the SOP for future assessment. An automated biochemical analyzer (Roche, Cobas c311) was used to obtain clinical features including fasting plasma glucose (FPG), HbA1c, and routine lipid profiles such as low-density lipoprotein cholesterol (LDL-C), high-density lipoprotein cholesterol (HDL-C), total cholesterol (TC), and triglyceride (TG).

### Determination of plasma ACars levels

Plasma ACars were carefully profiled by an experienced, professional technician from the central laboratory of Shanghai ProfLeader Biotech Co., China. The details of metabolomics methods used can also be found in the Supplementary Materials. In short, the thawed plasma samples were added with internal standard, methanol and methyl tert-butyl ether (MTBE), etc., and then vortex shaken, ice-water ultrasonic bath, rested, centrifuged, and dried before the assessment by ultra-performance liquid chromatography-electrospray ionization-tandem mass spectrometry (UPLC-ESI-MS/MS) system. To avoid or decrease potential information bias as much as possible, all the following procedures associated with the determinations were finished strictly following the SOP by a single technician, and professional quality control measures were also applied. Samples from the cases and controls were processed in a random manner, and the technician was also blind to the categories of these samples. Plasma fractions were separated by the UPLC-ESI-MS/MS and identified by their retention times compared with those internal standards and expressed as peak areas.

In addition, the quantitative analyses of the screened plasma ACars were performed under the same conditions as above, and their intensities were calculated by plotting the peak area of the corresponding substance against the concentration of the standard (μmol/L) using a linear calibration curve.

### Sample size estimation

To ensure that the sample size was sufficient to meet statistical requirements and to ensure the reliability of our findings, we estimated the sample size using G*Power (http://stats.idre.ucla.edu/other/gpower/). With type I error as 0.05, effect size equals to 0.8, a total sample size of 60 will be needed to achieve a power of 0.8 at the allocation ratio of 1 (**Supplementary Figure 2**).

### Statistical analysis

Considering that missing data may lead to some bias and affect the credibility of findings to some extent, multiple imputations were performed for all covariates with a missing proportion of less than 20%. Demographic and clinical characteristics of cases and controls were compared by Chi-square test (categorical variables) and Student’s t-test or Mann-Whitney U tests (continuous variables), respectively. Due to the skewed distributions of plasma ACars, we improved their normality first by log-transforming and normalizing them to Z scores. The Pearson correlation coefficients were used to describe the relationship between any two plasma ACars.

To comprehensively examine the association of plasma ACars with DR, the adjusted odds ratio (OR) and 95% confidence intervals (CI) were first estimated based on increases in each interquartile range (IQR) of ACars, depending on a multiple logistic regression model with adjusting for age, sex, BMI, smoking habits, alcohol consumption, education, duration of diabetes, TG, FPG, systolic blood pressure (SBP) and study center. In addition, considering that the relationship between ACars and DR may vary depending on their acyl chain length and unsaturation degree, ACars were classified into two categories, medium and long chains (defined as having carbon chains of 7 to 14, and ≥16, respectively), and further analyzed based on carbon atom and double bond numbers.

To thoroughly assess the potential role of ACars in the detection of DR, some commonly used feature selection approaches including least absolute shrinkage and selection operator (LASSO), elastic net (ENET), and weighted quantile sum (WQS) regression models were applied to detect potential biomarkers of DR from plasma ACars. Concentrations of the screened ACars were further determined based on UPLC-ESI-MS/MS platform. The relationship between potential biomarkers and DR was described in two ways: with concentrations as continuous variables [scaled to interquartile range (IQR)] and as categorical variables (tertiles). Meanwhile, linear trend tests were also performed. Restricted cubic spline (RCS) regression models with three knots of 5%, 50%, and 95% were additionally performed to further deeply investigate the potential dose-response relationships between detected biomarkers and the odds of DR. In addition, subgroup analyses and sensitivity analyses were also applied to test reliability and stability of the association.

To evaluate the role of screened plasma ACars in DR identification, we compared the performance of these potential biomarkers within models constructed from traditional indicators such as smoking habits, alcohol consumption, education, SBP, TG, FPG, and duration of diabetes by area under receiver operating characteristic (ROC) curves (AUC), sensitivity, specificity, positive predictive value (PPV), and negative predictive value (NPV). The comparisons of AUCs were additionally conducted by Delong’s tests.

All statistical analyses were conducted using Stata/MP 15.1 for windows (Copyright 1985-2017 Stata Corp LLC, College Station, Texas 77845, USA) and R version 4.0.4 for Windows (R Foundation for Statistical Computing, Vienna, Austria). The R package gWQS were used for WQS analyses (20). Two-tailed *P* values ≤ 0.05 were regarded as statistically significant.

## Results

### Characteristics of study participants

Characteristics of 138 participants were presented in **Table 1**. In addition to age, sex, BMI, BP, and HbA1c matched under PSM, fasting HDL, LDL, FPG, TG, TC, and lifestyle factors including smoking habits, alcohol consumption, occupation, and education status in the cases and controls were also balanced and comparable. However, patients with DR were more likely to have longer durations of diabetes than those without DR.

**Table 1:** Demographic and clinical characteristics between DM and DR subjects.

Variables	DM (n=69)	DR (n=69)	*P* values
Age, years	54.0 (49.0, 65.0)	57.0 (51.0, 65.0)	0.107
Gender (%)			0.863
Male	40 (58.0)	39 (56.5)	
Female	29 (42.0)	30 (43.5)	
BMI, kg/m2	24.10 (22.07, 26.61)	24.14 (22.49, 26.78)	0.609
DBP, mmHg	79 (75, 87)	77 (70, 81)	0.126
SBP, mmHg	126 (119, 140)	134 (121, 146)	0.077
FPG, mmol/L	8.12 (6.59, 10.80)	8.50 (6.31, 10.89)	0.893
HbA1c, %	9.94 ± 2.08	9.68 ± 1.92	0.433
HDL, mmol/L	1.02 (0.81, 1.35)	1.09 (0.86, 1.24)	0.946
LDL, mmol/L	2.62 ± 0.97	2.52 ± 1.06	0.562
TG, mmol/L	1.56 (1.03, 1.92)	1.41 (1.00, 1.93)	0.488
TC, mmol/L	4.72 ± 1.14	4.54 ± 1.39	0.424
Duration of diabetes, years	8.92 ± 6.21	12.43 ± 6.83	0.002
Education (%)			0.283
Junior high school or below	53 (76.8)	58 (84.1)	
High school or above	16 (23.2)	11 (15.9)	
Occupation (%)			0.390
Manual workers	31 (44.9)	36 (52.2)	
Mental workers	21 (30.4)	14 (20.3)	
Both	17 (24.6)	19 (27.5)	
Smoking habits (%)			0.700
Nonsmoker	41 (59.4)	38 (55.1)	
Current smoker	22 (31.9)	22 (31.9)	
Former smoker	6 (8.7)	9 (13.0)	
Alcohol consumption (%)			0.160
Nondrinkers	36 (52.2)	32 (46.4)	
Current drinkers	29 (42.0)	26 (37.7)	
Former drinkers	4 (5.8)	11 (15.9)	
Center (%)			0.106
Wenzhou	41 (59.4)	50 (72.5)	
Anhui	28 (40.6)	19 (27.5)	

Data are presented as mean ± SD for normal distributed variables, median (1st quartile, 3rd quartile) for skewed distributed variables, number (%) for categorical variables. When comparing differences between groups, the Chi-square test is used for categorical variables, the Student’s t-test for normally distributed variables, and t Mann-Whitney U tests for skewed variables.

DM, type 2 diabetes (T2D) without diabetic retinopathy; DR, T2D with diabetic retinopathy.

### Distributions of plasma ACars

In the present study, we detected a panel of 20 plasma ACars that span the full range of total carbon atomic number (C8-C26) and unsaturation degree (n[C=C]=0–4) (**Supplementary Table 1**). The distribution of these ACars levels were shown in **Supplementary Figure 3**. The cases would like to have lower levels of ACars (8:0, 11:1, 12:0, 14:1, 16:0, 16:1, 16:2, 18:1, and 26:1) than the controls (*P* < 0.05). Except for two pairs of ACars (13:0 & 20:1, 13:0 & 26:1), the correlation coefficients among other ACars showed significant positive relationships between any two of them (r > 0, *P* ≤ 0.05) (**Supplementary Figure 4**).

### Plasma ACars profiles and DR

**Figure 1:**
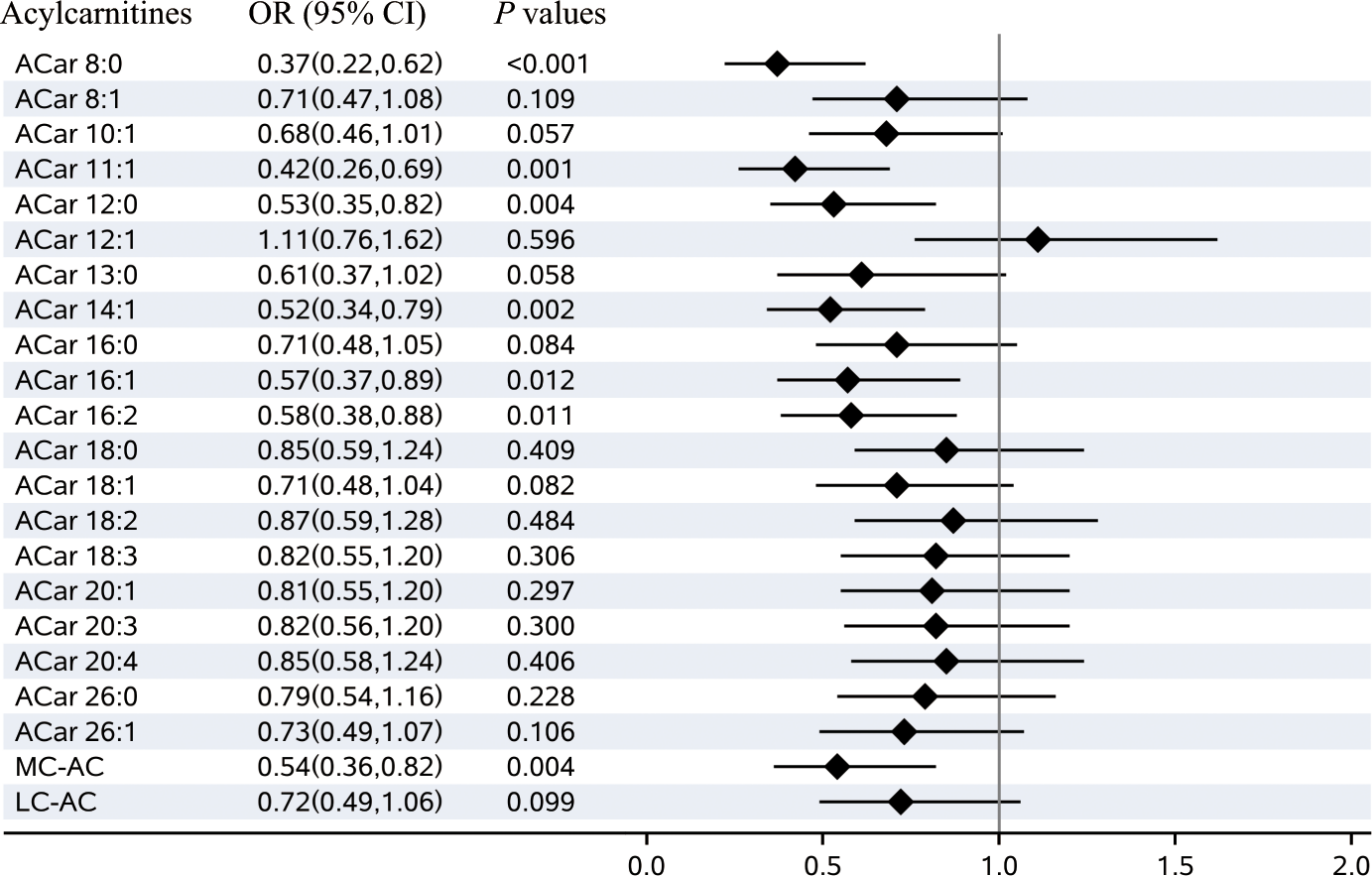
Plasma acylcarnitines and the risks of diabetic retinopathy. Notes: Odds ratio (OR) of developing diabetic retinopathy per SD increase in each plasma acylcarnitines were calculated by the logistic regression model, after adjusting for age, sex, BMI, smoking habits, alcohol consumption, education, duration of diabetes, TG, FPG, SBP, and center. OR, odds ratio; CI, confidence interval; MC-AC, medium-chain acylcarnitines; LC-AC, long-chain acylcarnitines..

**Figure 2:**
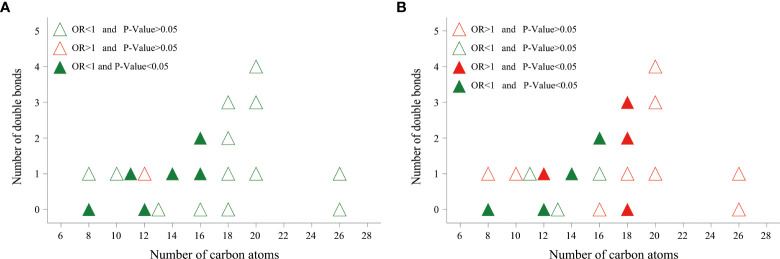
The relationship between DR risk and total number of carbon atoms and degree of saturation of various acylcarnitines. Odds ratio (OR) of developing DR per SD increase in each plasma acylcarnitines was calculated by logistic regression model, after adjusting for age, sex, BMI, smoking habits, alcohol consumption, education, duration of diabetes, TG, FPG, SBP, and center. **(A)** Acylcarnitines were log-transformed, and standardized before logistic regression analysis. **(B)** Acylcarnitines were divided by total acylcarnitine concentration, log-transformed, and standardized before logistic regression analysis. OR, odds ratio.

Based on multivariable logistic regression analyses, significant differences between the cases and controls were observed in 6 kinds of plasma ACars (8:0, 11:1, 12:0, 14:1, 16:1, and 16:2) after adjusting for age, gender, BMI, smoking habits, alcohol consumption, education, duration of diabetes, TG, FPG, SBP, and the study center (**Figure 1**). Meanwhile, restricted cubic spline (RCS) model-based results also revealed apparent non-linear dose-response relationships between the 6 plasma ACars and DR (all *P*_for nonlinearity_ > 0.05) (**Supplementary Figure 5**). In addition, a moderately low risk was observed for those with increased medium-chain ACars, whereas no association was found between long-chain ACars and DR (**Figure 1**). Similar results were also detected in the association between ACars and DR when they were stratified by different carbon atom numbers and double bonds. We found that ACars with lower carbon atomic numbers (C ≤ 16) were negatively associated with the odds of DR, regardless of whether the total ACars were adjusted or not (**Figures 2A, B**, green filled). However, interestingly, the association between ACars with higher carbon atom number (C18) and the likelihood of DR changed from no significant (*P* > 0.05) (**Figure 2A**, green unfilled) to significantly positive (*P* ≤ 0.05) (**Figure 2B**, red filled) after adjusting for total ACars.

### Association of ACar 8:0 with DR and its role in the identification of DR

**Figure 3:**
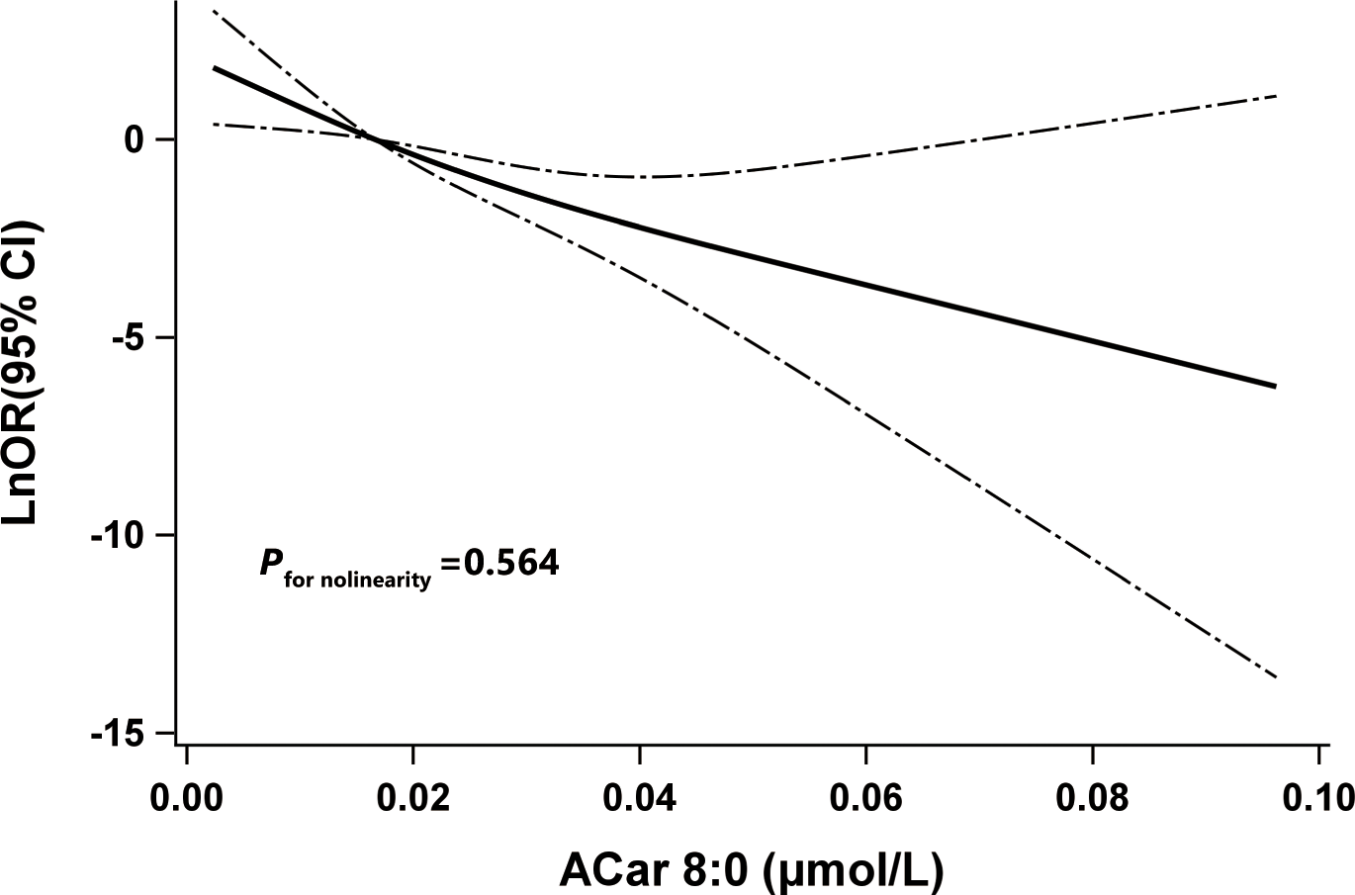
The restricted cubic spline for the association between Acar 8:0 and odds ratio (natural log-transformed) of DR. Notes: Knots were located at the 5th, 50th, and 95th percentiles of Acar 8:0.The solid line indicates LnOR and dashed lines indicate 95% CI. Adjusted confounders were age, sex, BMI, smoking habits, alcohol consumption, education, duration of diabetes, TG, FPG, SBP, and center. P for nonlinearity was used to test for linearity. DR, type 2 diabetic patients with diabetic retinopathy; LnOR, natural log-transformed odds ratios; CI, confidence interval, ACar, acylcarnitine.

To avoid or greatly decrease bias induced by potential collinearity and overfitting before constructing reasonable multiple regression models, several commonly used approaches containing LASSO, ENET and WQS regression models were respectively conducted to screen valuable ACars. Among all 20 detected plasma ACars, only ACar 8:0 was selected by LASSO and ENET and had the highest weight in the WQS model (**Supplementary Table 2**). To accurately clarify the relationship between plasma ACar 8:0 and DR, its actual concentration was determined by a calibration curve (**Supplementary Figure 6**). As was shown in **Table 2**, with per IQR increase of ACar 8:0, the adjusted likelihood of DR significantly decreased by 70% (OR: 0.30, 95% CI: 0.17 - 0.55). Similar results were also observed when evaluating the association *via* a quantile regression model: comparing to subjects with the lowest tertile plasma ACar 8:0, the adjusted odds of DR decreased by 78% (OR: 0.22, 95% CI: 0.08 - 0.59) and 88% (OR: 0.12, 95% CI: 0.04 - 0.36) for those in the 2^nd^ and 3^rd^ tertile of ACar 8:0, respectively. An apparent linear trend of ACar 8:0 with DR was additionally detected (*P*_for trend_ < 0.001). And results based on further sensitivity analyses (**Supplementary Table 4**) as well as RCS model (**Figure 3**) were highly consistent. None of the other variables, including age, gender, blood pressure, and regions showed significant effect modification on the association between plasma ACar 8:0 and the risk of DR (P for interaction > 0.05 for all these stratified variables) (**Supplementary Figure 7**). And patients with increasing levels of DR did not have decreasingly lower ACar 8:0 levels (**Supplementary Figure 8**).

**Table 2:** Association between ACar 8:0 and the presence of DR.

ACar 8:0 (μmol/L)	N	Cases (%)	Model 1 ^a^		Model 2 ^b^		Model 3 ^c^	
			OR (95%CI)	*P* values	OR (95%CI)	*P* values	OR (95%CI)	*P* values
Per IQR=0.012	138	69(50.00)	0.33(0.20,0.55)	<0.001	0.30(0.17,0.54)	<0.001	0.30(0.17,0.55)	<0.001
Tertiles								
T_1_ (0.002~)	46	35(76.09)	1.00(1.00,1.00)	Ref.	1.00(1.00,1.00)	Ref.	1.00(1.00,1.00)	Ref.
T_2_ (0.014~)	46	20(43.48)	0.24(0.10,0.59)	0.002	0.21(0.08,0.55)	0.002	0.22(0.08,0.59)	0.003
T_3_ (0.021~0.097)	46	14(30.43)	0.14(0.06,0.35)	<0.001	0.11(0.04,0.33)	<0.001	0.12(0.04,0.36)	<0.001
*P _for_*_trend_				<0.001		<0.001		<0.001

^a^Unadjusted for potential confounders; ^b^Adjusted for age, sex, BMI, smoking habits, alcohol consumption, education, TG, FPG, SBP, and center; ^c^Adjusted for age, sex, BMI, smoking habits, alcohol consumption, education, TG, FPG, SBP, center, duration of diabetes.

DR, type 2 diabetic patients with diabetic retinopathy; n, numbers of subjects in each stratum; Case (%), numbers with DR and percentage; OR, odds ratio; CI, confidence interval; T1, T2, and T3, the 1st, 2nd, and 3rd tertiles of Acar 8:0, respectively, ACar, acylcarnitine.

We further explored the potential role of ACar 8:0 as a biomarker for early DR identification. Based on ROC curve analyses, with removing matching factors from the reference model, combining ACar 8:0 with the reference (Ref) model increased the area under the curve (AUC) from 0.67 (0.58, 0.76) (Ref) to 0.77 (0.70, 0.85) (Ref + ACar 8:0) (P = 0.011) (**Supplementary Table 5**; **Supplementary Figure 9**). Meanwhile, the sensitivity, specificity, positive predictive value (PPV), and negative predictive value (NPV) of the combined model were 82.61%, 65.22%, 70.37%, and 78.95%, respectively (**Supplementary Table 5**).

## Discussion

In this PSM-based case-control study, we found that four plasma ACars (8:0, 12:0, 14:1 and 16:2), mainly medium-chain ACars, were inversely associated with the risk of DR, while four other ACars (12:1, 18:0, 18:2 and 18:3), predominantly long-chain ACars, were positively associated with DR after adjusting for total ACars. Particularly, the magnitude of the association between ACar 8:0 and DR was substantial, with high ACar 8:0 levels associated with an approximately 80% lower odds ratio of DR across tertiles in the categorical analysis. And the association did not seem to differ by age, gender, BMI, blood pressure, and variation in regions. In addition, ACars 8:0 might also can serve as an ideal biomarker for DR identification though its alteration was not significantly observed amongst different stages of DR.

Available evidence suggests that ACars are associated with β-oxidative dysfunction, mitochondrial stress, and insulin resistance (9), and a panel of ACars, especially those with long chains, are significantly associated with increased risks of type 2 diabetes (21, 22). However, studies on the relationship between ACars and DR remain scarce and controversial. Wang et al. (14) found that individuals with DR were more likely to had higher levels of plasma Acars than those without DR. However, an untargeted metabolomics study showed that increased levels of ACars were related to the higher risk of DR (6). This was in line with supportive evidence from another study that patients with PDR had higher levels of ACars in vitreous compared with non-diabetic controls (23). Our current findings suggest that there may be potential beneficial (inverse) associations between medium-chain ACars and DR, but positive associations between long-chain ACars and DR. Although the discrepant findings of these studies might be ascribed to the differences in study design, study population, biosample type, and methodology associated with exposure estimation, the greatest differences may arise from the complex inter-relationships and metabolic effects of different ACars. Notably, in our study, after adjusting for the total ACars level, the odds ratio between majority of long-chain ACars and DR changed from less than 1 to greater than 1. Therefore, the negative association of long-chain ACars and DR in the previous study (14) should be interpreted with caution. In contrast, the inverse association between medium-chain ACars and the risk of DR is robust with or without correction of the total ACar level. The precise mechanisms of how ACars may influence DR risk are unknown, but there is evidence suggesting that incomplete oxidation of fatty acids was a major contributor to the pathway.

Biologically, ACars are mainly divided into short, medium and long chains according to the length of the carbon chain. Except for short-chain ACars, which are mainly derived from alternative energy sources, i.e., branched-chain amino acids (24), medium- and long-chains are predominantly originated from acylated derivatives of L-carnitine after β-oxidation of fatty acids (FAO) in mitochondria and peroxisomes (25). A previous study has reported that peroxisomal FAO is directly related to energy production (26). Because of this functional difference, the majority of medium- and long-chain ACars can be produced by mitochondrial FAO (27). As shown in **Supplementary Figure 10**, when FAO does not proceed completely (fewer cycles), it will inevitably lead to a reduction in the levels of medium-chain ACars, while long-chain ACars may produce a buildup (27). Therefore, the accumulations of long-chain ACars in plasma are often used to suggest defective FAO. Although there is no direct evidence that long-chain ACars are harmful to DR, a previous study has suggested that the inability of patients to regulate the oxidation of long-chain fatty acids will make them more susceptible to oxidative stress (28), which further increases the risk of developing DR (29). Therefore, this may be a potential interpretation of why high levels of long-chain ACars in plasma are associated with DR, while medium-chain ACars showed negative associations.

Furthermore, our findings highly suggest that ACar 8:0 may serve as a potential biomarker in distinguishing DR from T2D. Depending on the evidence from other studies (30), it shows that plasma ACar 8:0 appears to have unique biological functions and can be used in clinical practice to detect diseases caused by impaired energy metabolism, particularly those caused by defects in mitochondrial medium -chain metabolism. Although extrapolating from possible mechanisms, such a result may suggest that T2D patients without DR can complete more cycles of FAO as compared to those with DR, further biochemical and clinical research is needed to examine a causal role of FAO in glycemic outcomes, particularly in diabetes complications.

The strengths of this study can be summarized as follows. First, the cases and controls are all enrolled in the endocrinology departments of comprehensive hospitals. Among participants, no one has obvious ocular symptoms, which may avoid some potential factors due to eye diseases in our findings. Second, the cases and controls are matched by the PSM approach, which has been widely accepted as an optimal strategy to effectively adjust for some known or unknown confounding bias in observational studies and can achieve class-balanced data. Third, the study population was enrolled from the departments of endocrinology in two top general hospitals in Zhejiang and Anhui, China. The two provinces cover a population of over 150 million residents, which may be beneficial to largely improve the credibility of our findings. In addition, LASSO, ENET, and WQS regression models are simultaneously used to screen the desirable profile of plasma ACars, which can not only avoid potential multicollinearity in the following multiple regression models but also efficiently and reasonably assess the effects of several ACars at the same time.

Inevitably, this study also has limitations. First, our findings are mainly based on a case-control study, which cannot exactly interpret possible causal relationships and mechanisms between ACars and DR. Although our results need to be additionally confirmed by longitudinal studies, their credibility has been demonstrated to a large extent depending on the results of sensitivity analyses. Second, although our sample size may be relatively small, it does not necessarily equal inadequacy. As is shown in **Supplementary Figure 1**, a total sample size over 60 will lead to power over 0.8 and the current sample size is sufficient to meet statistical requirements since the associated power has achieved 0.998, which ensures that our findings are credible. Third, diet patterns may partly affect the modification of plasma ACar. Third, data related to the red meat diet was not collected in the present study, but changes in the subjects’ daily diet should not significantly affect the ACars concentrations measured in fasted blood samples, which could be used as a marker for long-term *in vivo* ACars levels (31). Fourth, as the PSM approach was applied, matched factors were not used in the construction of the identification model, but the model also performed well when it contained only ACar 8:0. Fifth, participants were limited to those who were from Zhejiang and Anhui, which might restrict the generalization of our findings, but these two places are representative of the areas in eastern China and the findings are consistent between the two areas.

## Conclusions

Plasma ACar 8:0 is significantly associated with decreased risks of DR in T2D patients and plays an important role in distinguishing DR patients from T2D subjects. Our findings may be helpful for the secondary prevention of DR in clinical practice and provide new insights into the mechanisms of DR as well as the possibility of new treatment target development.

## Data availability statement

The raw data supporting the conclusions of this article will be made available by the authors, without undue reservation.

## Ethics statement

The studies involving human participants were reviewed and approved by Ethics Committee of the Eye hospital of Wenzhou medical university (Number: KYK (2017) 46). The patients/participants provided their written informed consent to participate in this study.

## Author contributions

DJ and SZ conducted the study design, data management, data analysis and manuscript draft. HL, RH, JZ, and ML performed the quality control, data cleaning and analysis. ZX MC, YW, ZZ, and HW contributed to the epidemiological investigation, sample handling, data management and analysis. GM and CZ designed the study, thoroughly reviewed and edited the manuscript. All authors contributed to the manuscript revision, and approved the final version of manuscript. The corresponding authors had full access to all the data in the study and had final responsibility for the decision to submit for publication.

## Funding

This study was supported by the National key research plan (2020YFC2008201), National Nature Science Foundation of China (82070833), Zhejiang Basic Public Welfare Research Project (LGF19H260011), Natural Science Foundation of Zhejiang Province (LZ19H020001) and the Scientific and technological innovation activity (new talent) plan for college students in Zhejiang Province (2021R413062). Part of this work was also funded by Zhejiang Provincial Key Research & Development Program (2021C03070).

## Acknowledgments

We sincerely thank all participants who participated in this study and the team members from Wenzhou Medical University, Anhui Medical University, and Zhejiang University School of Medicine who assisted in the collection of samples. We are also grateful for the dedication and hard work of Shanghai ProfLeader Biotech Co., Ltd, for their careful assay of all the plasma samples.

## Conflict of interest

The authors declare that the research was conducted in the absence of any commercial or financial relationships that could be construed as a potential conflict of interest.

## Publisher’s note

All claims expressed in this article are solely those of the authors and do not necessarily represent those of their affiliated organizations, or those of the publisher, the editors and the reviewers. Any product that may be evaluated in this article, or claim that may be made by its manufacturer, is not guaranteed or endorsed by the publisher.
