# Supplementary material for: Plasma acylcarnitine and diabetic retinopathy: A study from Eastern China
Source: Front Endocrinol (Lausanne). 2022 Oct 27;13:977428. doi: 10.3389/fendo.2022.977428 (PMC9646944; doi:10.3389/fendo.2022.977428)
Supplement: Supplementary file 1 [file DataSheet_1.docx]

**Supplementary Materials**

Appendix 1: Supplementary Methods.

Appendix 2: Study flowchart. (**Supplementary Figure 1**)

Appendix 3: Power analysis. (**Supplementary Figure 2**)

Appendix 4: The comparison of plasma acylcarnitines between DM and DR. (**Supplementary Figure 3**)

Appendix 5: Heatmap of the correlations between 20 acylcarnitines among 138 participants. (**Supplementary Figure 4**)

Appendix 6: The restricted cubic spline for the association between plasma acylcarnitines and odds ratio (natural log-transformed) of DR. (**Supplementary Figure 5**)

Appendix 7: The calibration curve for the determination of ACar 8:0 concentration. (**Supplementary Figure 6**)

Appendix 8: Subgroup analyses on the presence of DR with Acar 8:0. (**Supplementary Figure 7**)

Appendix 9: The comparison of Acar 8:0 amongst DM and different DR statuses. (**Supplementary Figure 8**)

Appendix 10: ROC curves and AUC showing the discrimination ability of ACar 8:0 to detect DR. (**Supplementary Figure 9**)

Appendix 11: Fatty acid β-oxidation and acylcarnitine metabolic pathway. (**Supplementary Figure 10**)

Appendix 12: The chemical molecular structure of plasma acylcarnitines. (**Supplementary Table 1**)

Appendix 13: LASSO, ENET, WQS models to investigate the associations of plasma acylcarnitines with diabetic retinopathy. (**Supplementary Table 2**)

Appendix 14: Sensitivity analyses of the association between Acar 8:0 and the risk of DR. (**Supplementary Table 3**)

Appendix 15: AUC and *P* values of DeLong's test of the prediction model of the Acar 8:0. (**Supplementary Table 4**)

**Supplementary Methods**

**1. Inclusion and exclusion criteria**

The inclusion criteria for type 2 diabetic (T2D) patients with DR and without DR in our study were as follows: (1) T2D; (2) ≥35 years old. Participants with following situation would be excluded: (1) any other eye diseases or history of eye surgery; (2) cancer, infectious disease, mental disorder, heart failure, severe hypertension (systolic blood pressure ≥180 mm Hg or diastolic blood pressure ≥110 mm Hg) and any other severe chronic systemic disease; (3) poor quality of fundus photographs, which were not clear for DR diagnosis. The inclusion and exclusion criteria for healthy controls were similar to those above but without diabetes. Only those following each of the inclusion criteria and none of the exclusion criteria were potential participants.

**2. Details of grading DR**

Professional photographers used a digital non-mydriatic fundus camera to obtain bilateral retinal 45-degree images of the macula (centered on the fovea). Fundus photographs were graded preliminarily for the level of DR and other fundus lesions by two graders depending on the retinal photograph assessment(1). If one eye was unavailable for the classification, the other one was graded. The severity of DR was determined depending on the worse eye, and each eye was assessed as follows: no apparent and any DR, which will be further classified into mild nonproliferative DR (NPDR), moderate NPDR, severe NPDR, and PDR according to International Clinical Diabetic Retinopathy Disease Severity Scale (2). As DR was diagnosed by two independent ophthalmologists, it is needed to assess the inter-grader agreement. In this study, the kappa value calculated for the graders’ agreement on fundus photographs were 0.86. The disagreement samples were graded again by the director of our fundus reading center.

**3. Propensity Score Matching (PSM)**

Propensity scores are being used in observational studies to reduce bias. In the current study, we used case-control matching on the propensity score. Briefly, controls were chosen using propensity score matching using logistic models including age, gender, BMI, blood pressure and HbA1c. We used 1:1 matching to perform multivariate logistic regression to calculate and save the predicted probability of the dependent variable, the propensity score, for each observation in the data set. Once a match is made, the match is not reconsidered. That match is the best match currently available. The algorithm makes "best" matches first and "next-best" matches next, in a hierarchical sequence until no more matches can be made. Best matches are those with the highest digit match on propensity score^(3)^.

**4. Power analysis**

The present study is a continuous study of our previous work, in which the sample size has been carefully estimated. However, this does not mean that the previous sample size is necessarily suitable for this study, so we conducted a power analysis for this study to ensure that this study has sufficient statistical power to avoid false negative results and to increase the credibility of the study results as much as possible. We performed a post hoc power analysis depending on the available data to decrease the potential bias. The power analysis was calculated by G*Power version 3.1.9.2 (<http://stats.idre.ucla.edu/>other/gpower/), a statistical power analysis software proposed by scientists from University of California, Los Angeles. With type I error as 0.05, total sample size as 138 and two-sided test, the powers associated with Acar 8:0 on DR were greater than 0.99 using the effect size d = 0.8359167 calculated by the two-sample independent t test (Supplementary Figure 1A). In fact, as observed in Supplementary Figure 1B, if the effect size d=0.8, the power has exceeded 0.8 when the total sample content reaches about 60. In conclusion, depending on the results of above power analysis results, the current sample size, 69 pairs of DR vs. DM, was necessarily sufficient and well balance the power of tests.

**5.** **Details of Acylcarnitine Profile Measurements**

Plasma Acylcarnitines were assessed based on Ultra-High-Performance Liquid Chromatography-High Resolution Tandem Mass Spectrometry (UHPLC-HRMS/MS) platform by a professional technician. Acetonitrile, methanol, formate, ammonium formate and isopropanol in LC-MS grade (Optima®) were obtained from Fisher Scientific. Methyl tert-butyl ether (MTBE) and dichloromethane in HPLC grade (Ourchem®) were purchased from Sinopharm Chemical Reagent Co., Ltd (Shanghai, China). Acetonitrile, methanol, formate, ammonium formate and isopropanol in LC-MS grade (Optima®) were obtained from Fisher Scientific. Methyl tert-butyl ether (MTBE) and dichloromethane in HPLC grade (Ourchem®) were purchased from Sinopharm Chemical Reagent Co., Ltd (Shanghai, China). Ultra-High Performance Liquid Chromatography (UHPLC)–High Resolution Tandem Mass Spectrometry (HRMS/MS): ThermoFisher Ultimate 3000 UHPLC and ThermoFisher Q Exactive™ Hybrid Quadrupole-Orbitrap™ Mass Spectrometry (QE).Chromatographic Column: Waters ACQUITY UPLC® CSH™ C18 column (2.1mm × 100 mm, 1.7 μm).

To 40 μL thawed plasma sample, 20 μL internal standards (5 μg /mL of FA 18:0-d35, PC 15:0-18:1-d7, PE 15:0-18:1-d7, PG 15:0-18:1-d7, TAG 15:0-18:1-d7-15:0 and 2 μg /mL of LPC 18:1-d7, LPE 18:1-d7, SM d18:1/18:1-d9) and 300 μL methanol were added and vortexed 30s. Then, 1 mL MTBE (methyl tert-butyl ether) was subsequently supplemented. The mixtures were vortexed for 10s, ultrasonicated for 10 min. 300 μL deionized water was added, vortexed 30s and stood for 30 min at room temperature. After centrifugation at 5000 rcf for 10 min, 800 μL supernatant (MTBE phase) was evaporated to dryness. The residues were reconstituted in 100 μL dichloromethane/methanol (1:1, v/v) prior to perform UHPLC-HRMS/MS analysis. A total of 24 quality controls (QC) were prepared by pooling the plasma samples. One QC sample was injected after every ten study samples. Quality control (QC) sample was pooled from representative samples and analyzed with the same procedure as that for the experiment samples.

The eluents were analyzed on a ThermoFisher Q Exactive™ Hybrid Quadrupole-Orbitrap™ Mass Spectrometry (QE) in Heated Electrospray Ionization Positive (HESI+) and Negative (HESI-) mode, respectively. Spray voltage was set to 3.5kV for HESI+ and HESI-. Both Capillary and Aux Gas Temperature were 350 °C. Sheath gas flow rate was 40 (Arb). Aux gas flow rate was 10 (Arb). S-Lens RF Level was 50 (Arb). The full scan was operated at a high-resolution of 70000 FWHM (m/z=200) at a range of 130 - 1950 m/z with AGC Target setting at 1×106. Simultaneously, the fragment ions information of top 10 precursors each scan was acquired by Data-dependant acquisition (DDA) with HCD energy at 20, 30 and 40 eV, mass resolution of 17500 FWHM, and AGC Target of 5×105.

| 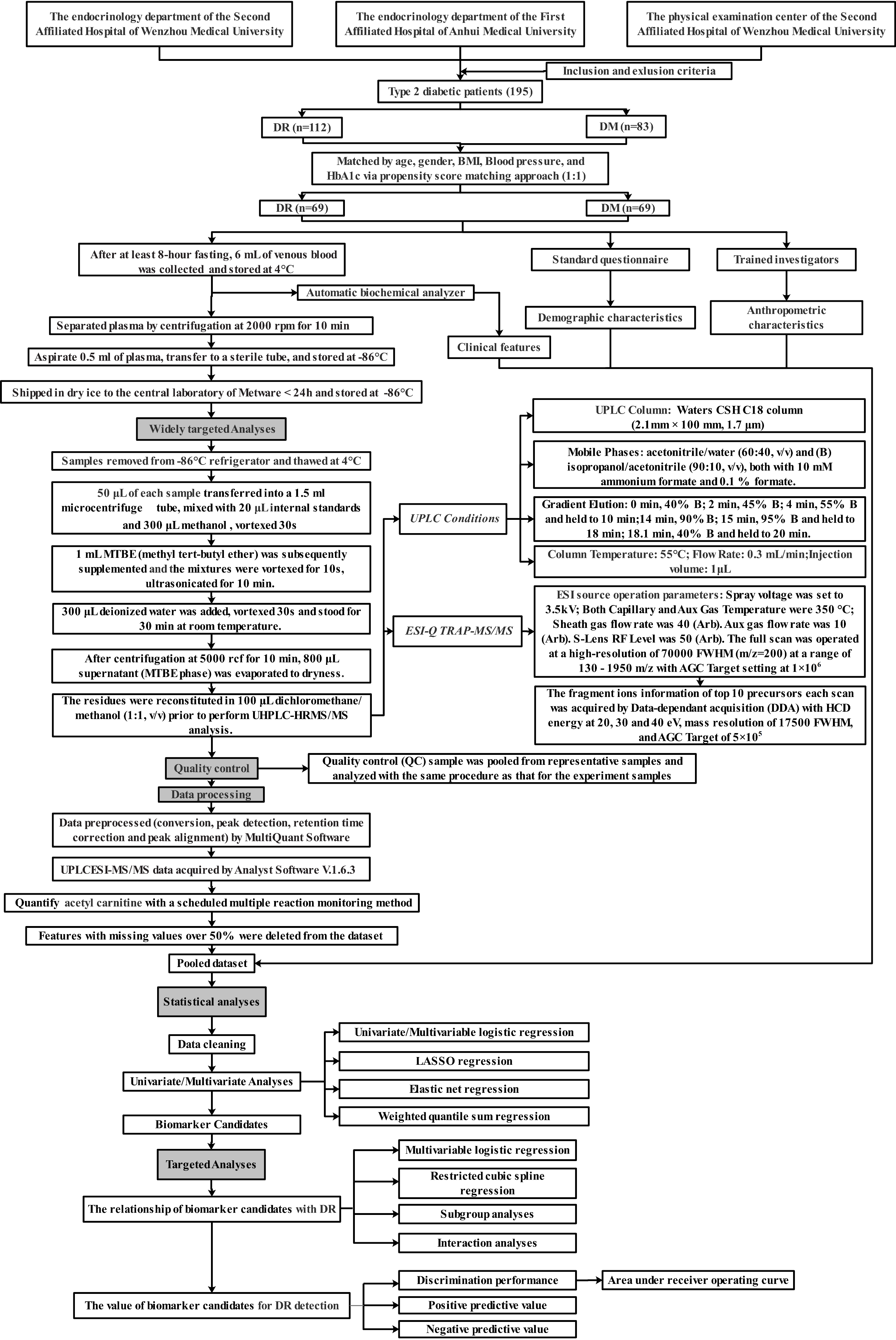 |
| --- |
| **Supplementary Figure 1. Study flowchart.**  ***Abbreviations:*** *DM: type 2 diabetic patients without diabetic retinopathy; DR: type 2 diabetic patients with diabetic retinopathy.* |

| 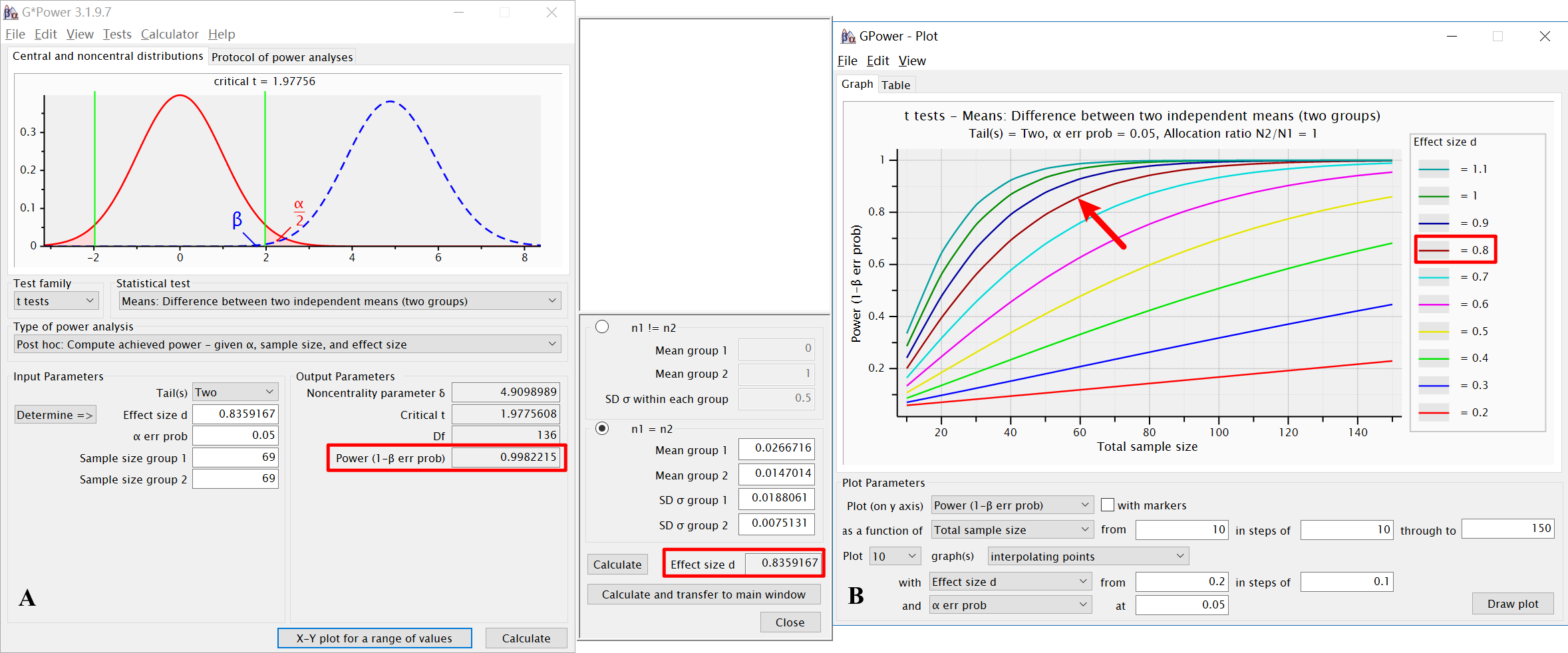 |
| --- |
| **Supplementary Figure 2. Power Analysis.** *A: calculation of effect size for power analysis. B: power analysis.* |

| 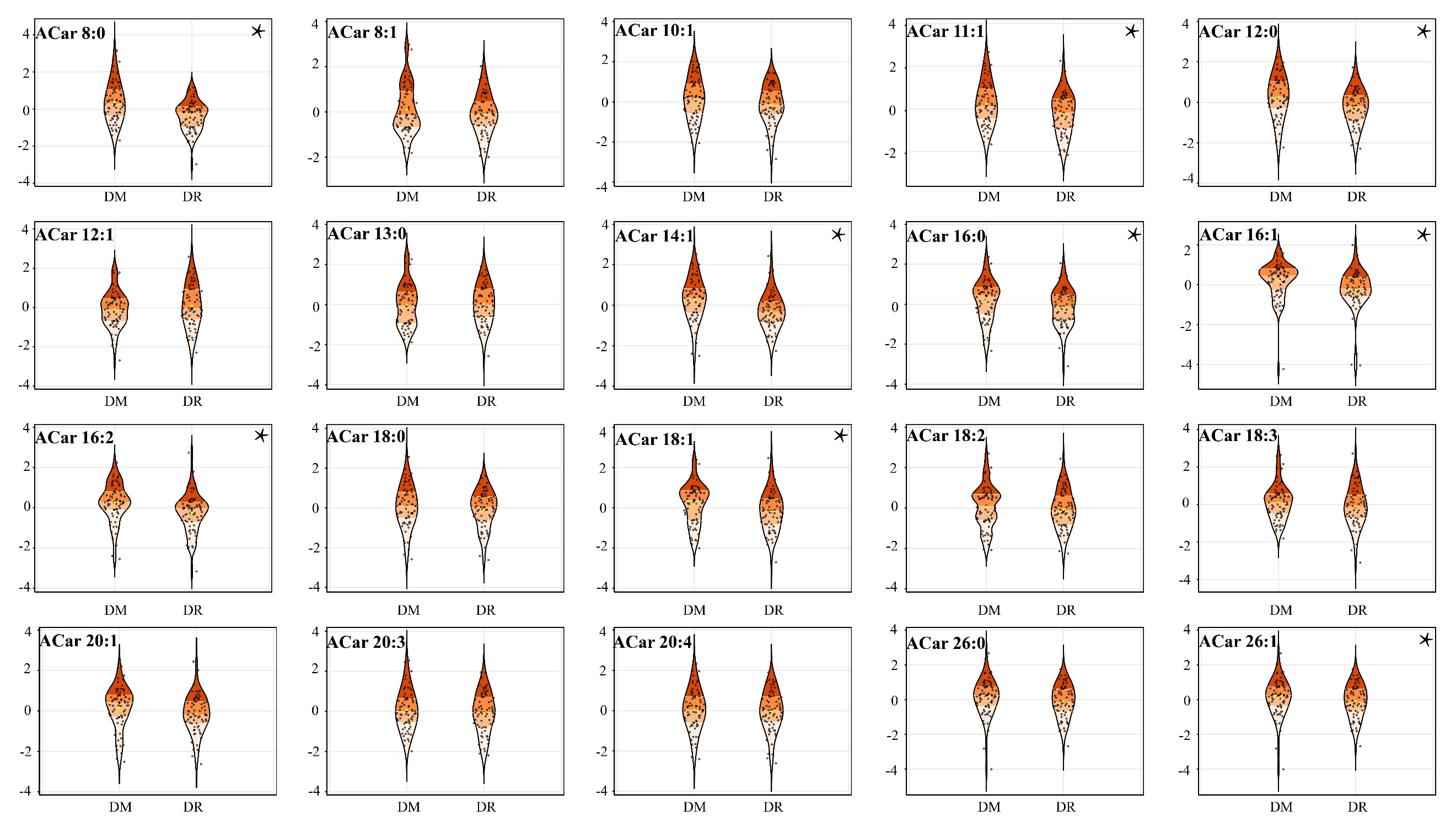 |
| --- |
| **Supplementary Figure 3. The comparison of plasma acylcarnitines between DM and DR.**  ***Notes:*** ** P value < 0.05.*  ***Abbreviations:*** *DM: type 2 diabetic patients without diabetic retinopathy; DR: type 2 diabetic patients with diabetic retinopathy.* |

| 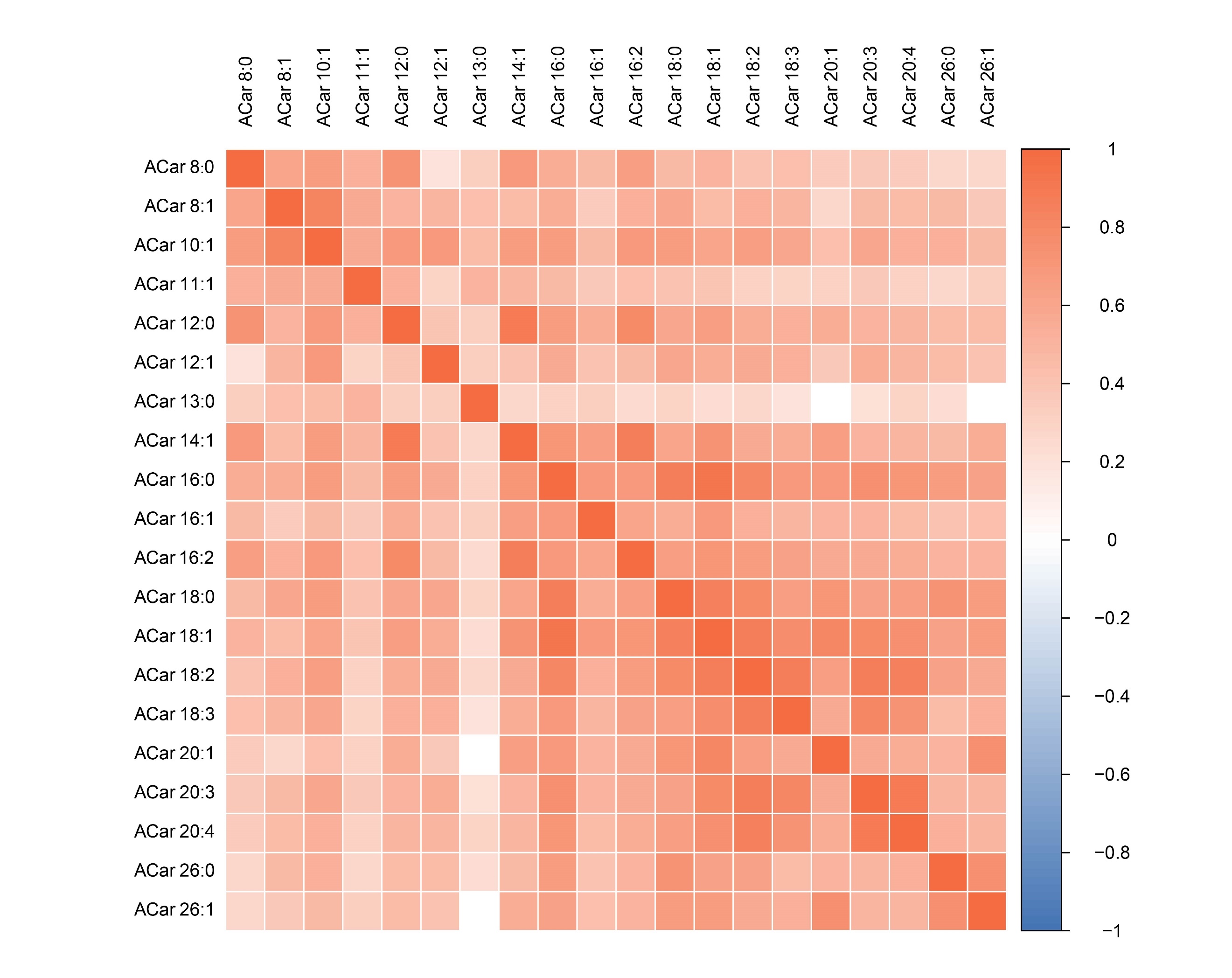 |
| --- |
| **Supplementary Figure 4. Heatmap of the correlations between 20 acylcarnitines among 138 participants.**  ***Notes:*** *The pearson correlation coefficients were used to characterize the correlation among different acylcarnitines; a darker color was associated with a greater correlation between the two acylcarnitines, while P > 0.05 of the pearson’s correlation between the two was shown in white.*  ***Abbreviations:*** *ACar: acylcarnitine.* |

| 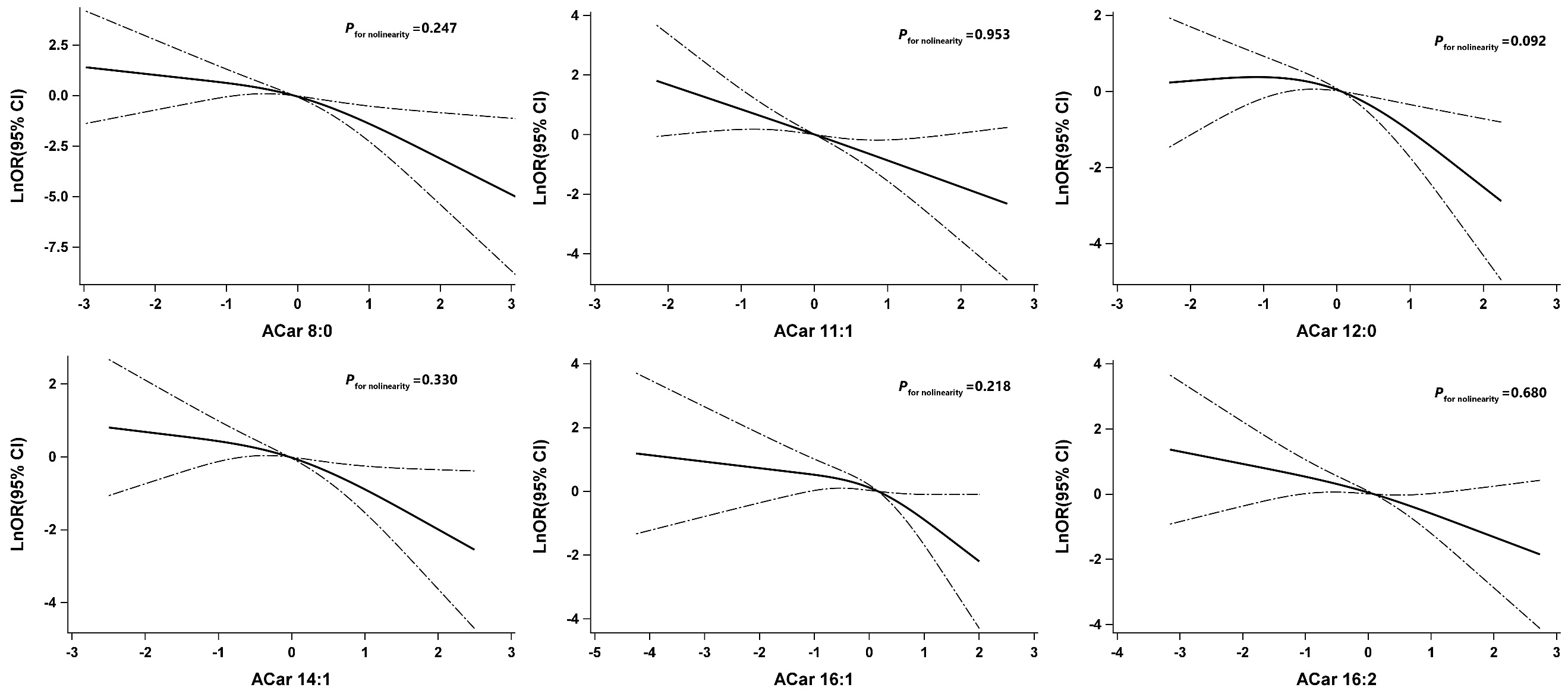 |
| --- |
| **Supplementary Figure 5. The restricted cubic spline for the association between plasma acylcarnitines and odds ratio (natural log-transformed) of DR.**  ***Notes:*** *Knots were located at the 5th, 50th, and 95th percentiles of* *plasma acylcarnitines; the solid line indicates LnOR and dashed lines indicate 95%CI. Adjusted confounders were age, sex, BMI, smoking habits, alcohol consumption, education, duration of diabetes, TG, FPG, SBP, and center. P _for nolinearity_ were used to test for linearity.*  ***Abbreviations:*** *DR: type 2 diabetic patients with diabetic retinopathy; LnOR: natural log-transformed odds ratios; CI: confidence interval, ACar: acylcarnitine.* |

|  |
| --- |
| **Supplementary Figure 6. The calibration curve for the determination of ACar 8:0 concentration.**  ***Notes:*** *The linear range was 0.0094-0.0375(μM); the Internal standards was LPC 18:1-d7.*  ** Regression coefficients were calculated for linearity ranging at concentrations mentioned above.* |

| 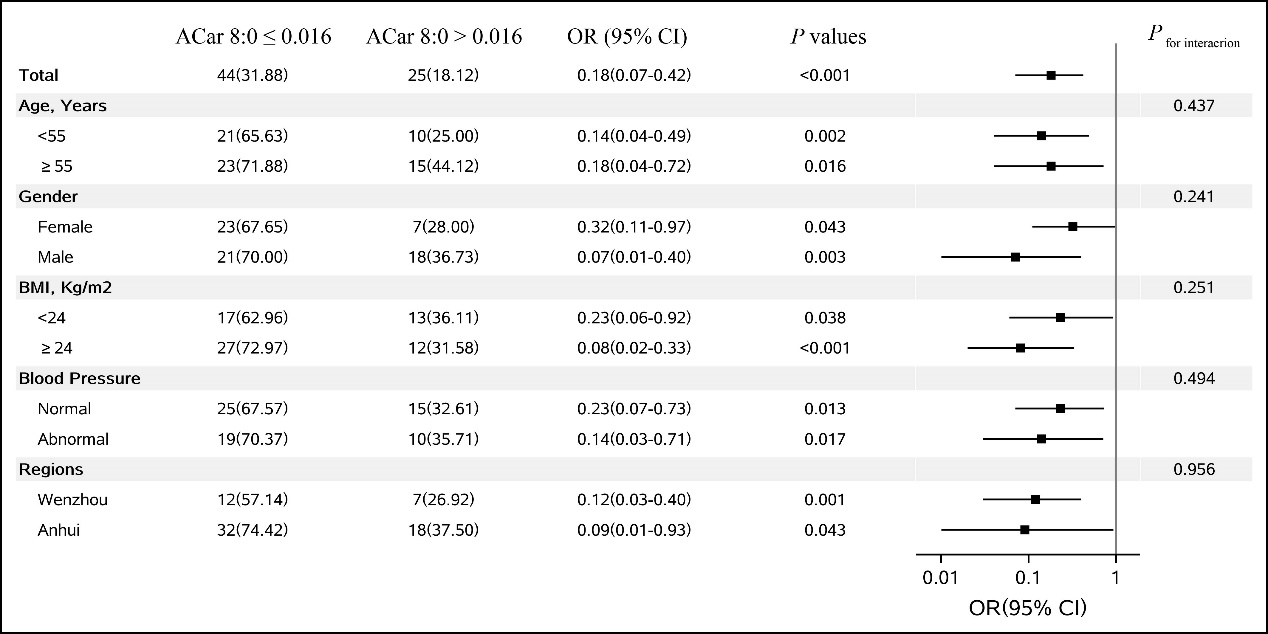 |
| --- |
| **Supplementary Figure 7. Subgroup analyses on the presence of DR with Acar 8:0.**  ***Notes:*** *Graphs show odds ratio (OR) and 95% confidence interval (CI) for the presence of diabetic retinopathy and Acar 8:0 adjusted for age, sex, BMI, smoking habits, alcohol consumption, education, duration of diabetes, TG, FPG, SBP, and center; 0.016 is the median of ACar 8:0; Blood Pressure normal is defined as systolic blood pressure <140 and diastolic blood pressure <90; P _for interaction_: P values for testing the interaction terms of stratified factors and ACar8:0 in logistic regression models.*  ***Abbreviations:*** *OR: odds ratio; CI: confidence interval, ACar: acylcarnitine.* |

| 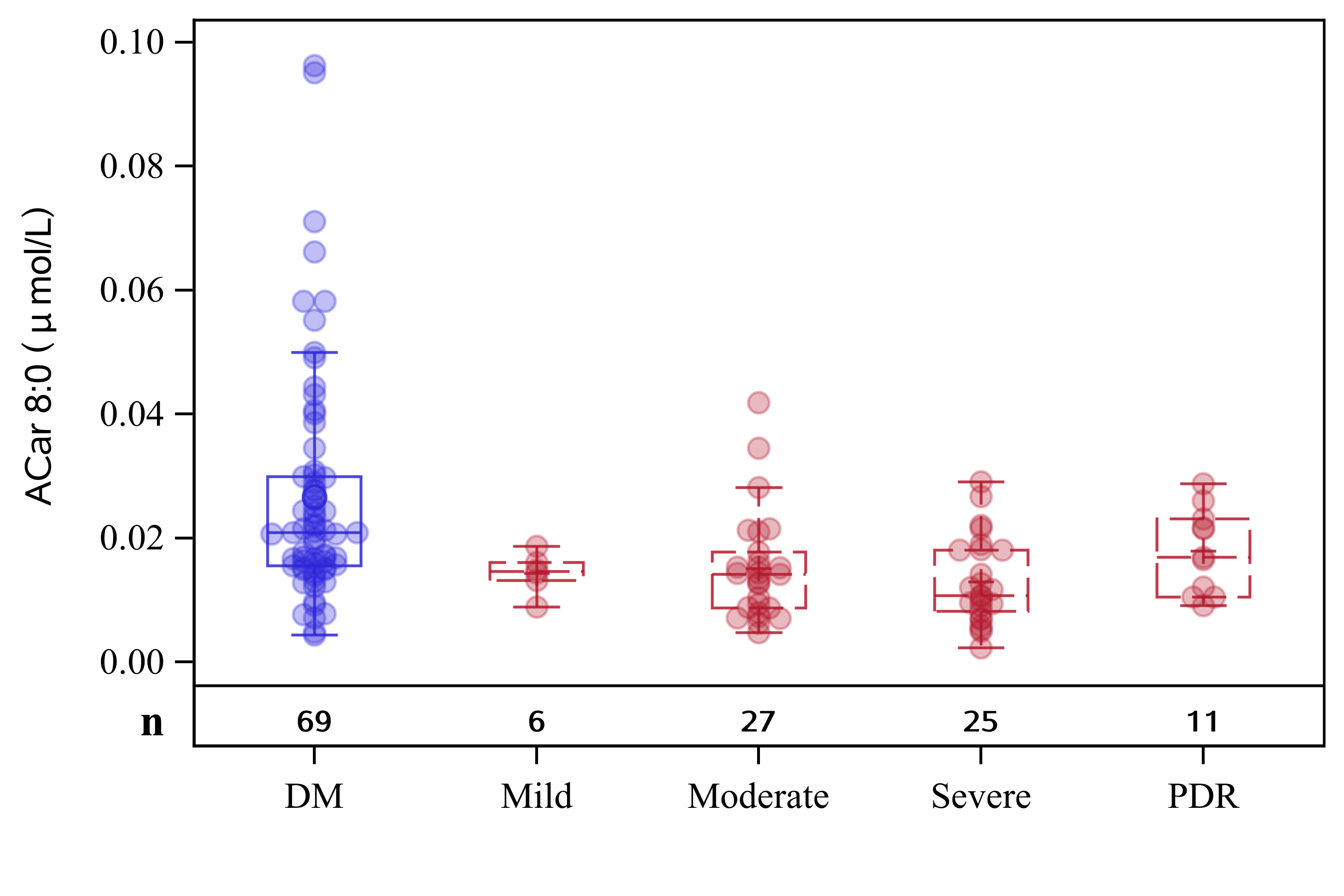 |
| --- |
| **Supplementary Figure 8. The Comparison of Acar 8:0 amongst DM and different DR statuses.**  ***Notes:*** *Mild, moderate, and severe indicate the degree of retinopathy in patients with nonproliferative diabetic retinopathy (NPDR), respectively; the numbers at the bottom of the graph indicate the number of patients at different stages of the disease.*  ***Abbreviations:*** *DM: type 2 diabetic patients without diabetic retinopathy; DR: type 2 diabetic patients with diabetic retinopathy; NPDR: nonproliferative DR; PDR: proliferative DR, ACar: acylcarnitine.* |

| 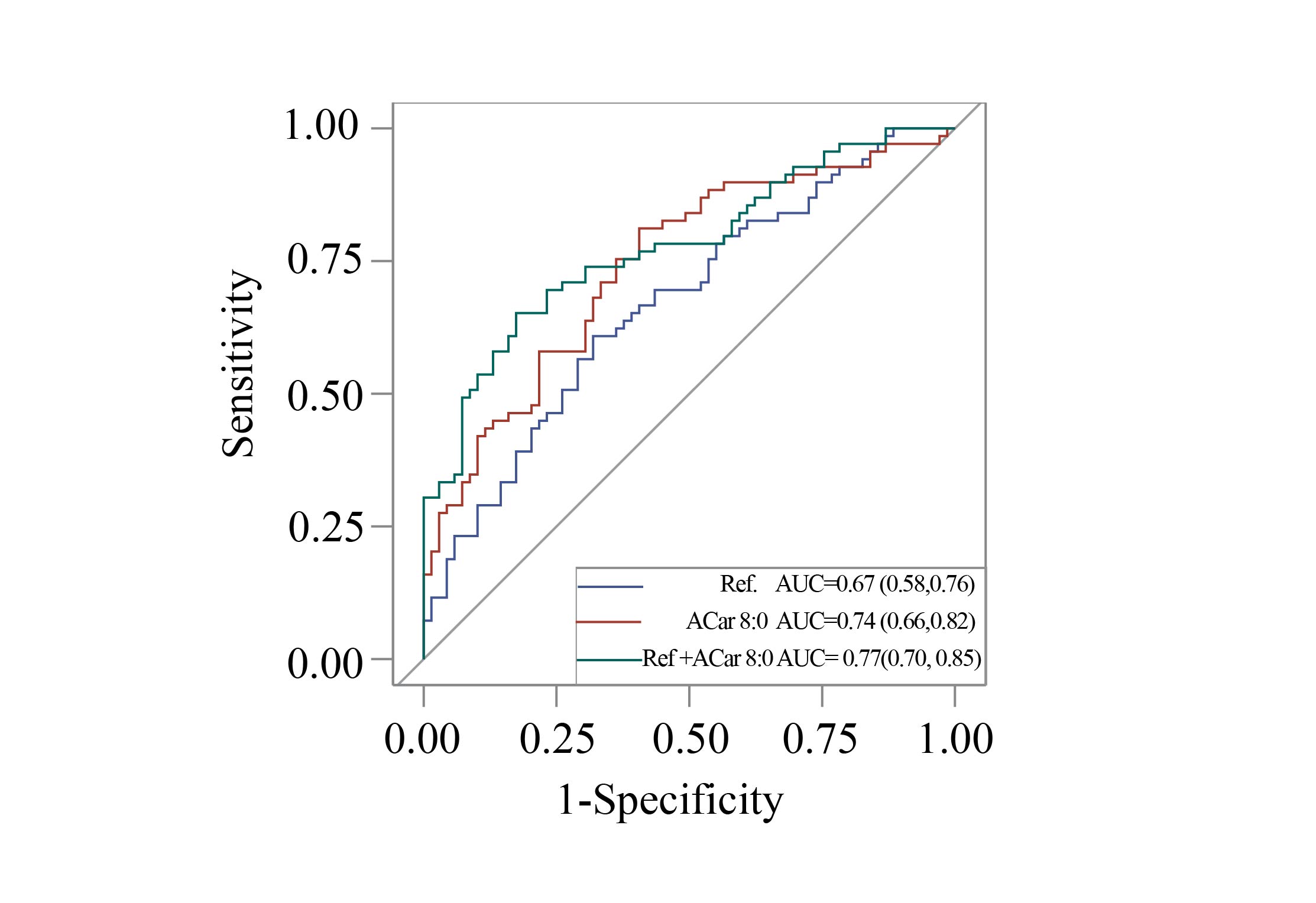 |
| --- |
| **Supplementary Figure 9. ROC curves and AUC showing the discrimination ability of ACar 8:0 to detect DR.**  ***Notes:*** *The reference model included smoking habits, alcohol consumption, education, SBP, TG, FPG, and duration of diabetes.*  ***Abbreviations:*** *DR: type 2 diabetic patients with diabetic retinopathy; ACar: acylcarnitine; ROC: receiver operating characteristic; AUC: areas under the ROC curves.* |

| ***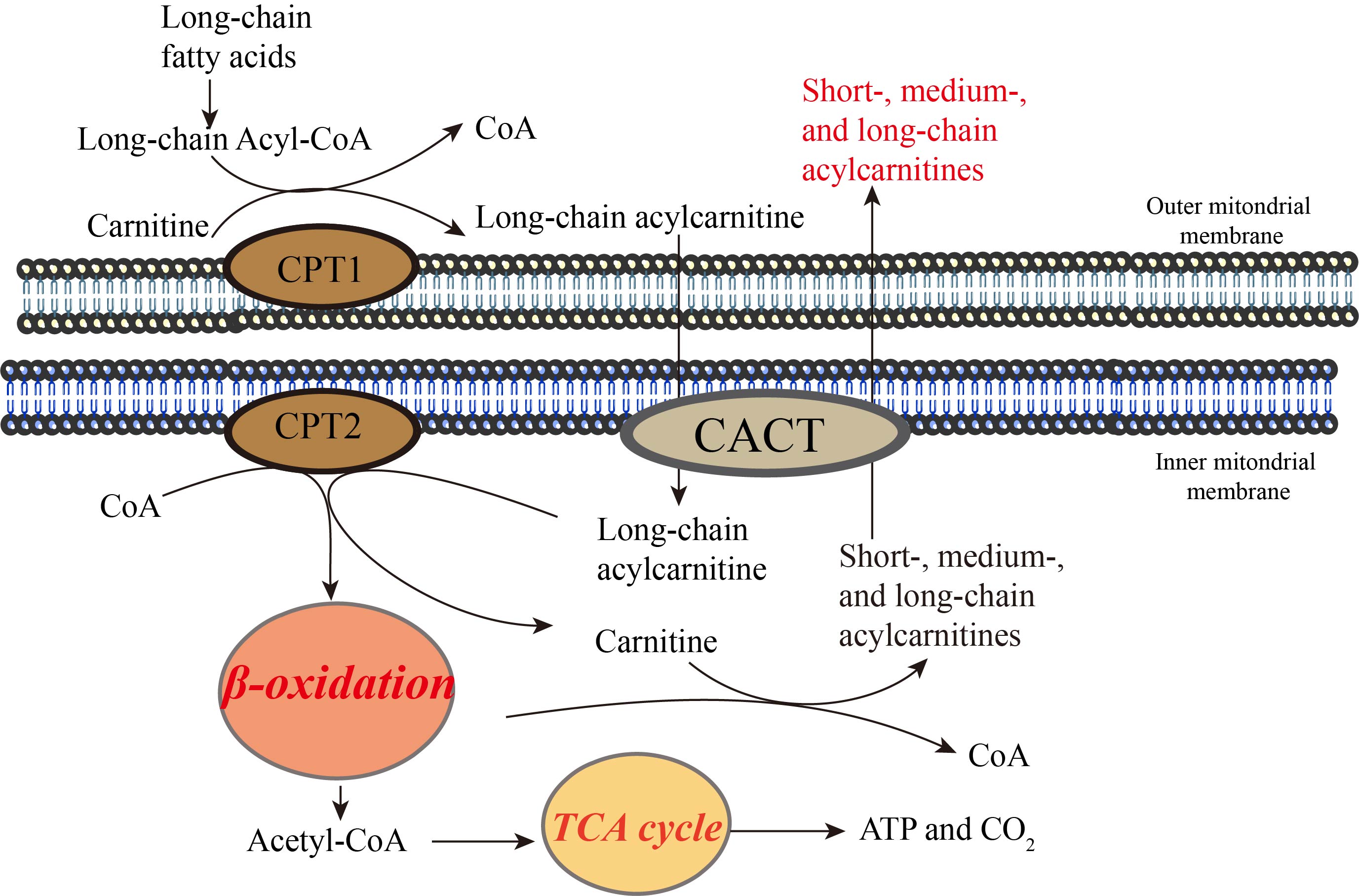*** |
| --- |
| **Supplementary Figure 10. Fatty acid β-oxidation and acylcarnitine metabolic pathway.** |

***Abbreviations:*** *CACT: carnitine/acylcarnitine translocase; CPT1: carnitine palmitoyltransferase 1; CPT2: carnitine palmitoyltransferase 2;TCA: tricarboxylic acid cycle; ATP:* *adenosine triphosphate.*

**Supplementary Table 1. The chemical molecular structure of plasma acylcarnitines.**

| **Acylcarnitines** | **InChIkeys** | **Molecular structure chemical formula** |
| --- | --- | --- |
| ACar 8:0 | CXTATJFJDMJMIY-UHFFFAOYNA-O | CCCCCCCC(=O)OC(CC(O)=O)C[N+](C)(C)C |
| ACar 8:1 | YAZIZQKDCOMBCP-BQYQJAHWNA-O | CCC\C=C\CCC(=O)OC(CC(O)=O)C[N+](C)(C)C |
| ACar 10:1 | OQWOHRPOYAVIOK-MDZDMXLPNA-O | CCCCC\C=C\CCC(=O)OC(CC(O)=O)C[N+](C)(C)C |
| ACar 11:1 | KTWZODJOQFVYRN-ZHACJKMWNA-O | CCCCCC\C=C\CCC(=O)OC(CC(O)=O)C[N+](C)(C)C |
| ACar 12:0 | FUJLYHJROOYKRA-UHFFFAOYNA-O | CCCCCCCCCCCC(=O)OC(CC(O)=O)C[N+](C)(C)C |
| ACar 12:1 | PETYOUXDCHQKTQ-ZHACJKMWNA-O | CCCCCC\C=C\CCCC(=O)OC(CC(O)=O)C[N+](C)(C)C |
| ACar 13:0 | OLAOWMDKXPIITQ-UHFFFAOYNA-O | CCCCCCCCCCCCC(=O)OC(CC(O)=O)C[N+](C)(C)C |
| ACar 14:1 | ABVVZYXTZLEOHP-CMDGGOBGNA-O | CCCC\C=C\CCCCCCCC(=O)OC(CC(O)=O)C[N+](C)(C)C |
| ACar 16:0 | XOMRRQXKHMYMOC-UHFFFAOYNA-O | CCCCCCCCCCCCCCCC(=O)OC(CC(O)=O)C[N+](C)(C)C |
| ACar 16:1 | NRCCZFJHTDUMBS-SEYXRHQNNA-O | CCCCCCCC\C=C/CCCCCC(=O)OC(CC(O)=O)C[N+](C)(C)C |
| ACar 16:2 | DPIJSJWXUXNTJD-OFXORYEXNA-O | CCCCCCCC\C=C/C\C=C\CCC(=O)OC(CC(O)=O)C[N+](C)(C)C |
| ACar 18:0 | FNPHNLNTJNMAEE-UHFFFAOYNA-O | CCCCCCCCCCCCCCCCCC(=O)OC(CC(O)=O)C[N+](C)(C)C |
| ACar 18:1 | HOAMADDCQBUDDY-KHPPLWFENA-O | CCCCCC\C=C/CCCCCCCCCC(=O)OC(CC(O)=O)C[N+](C)(C)C |
| ACar 18:2 | HQMPRWWWVKTZAS-HULFFUFUNA-O | CCCCC\C=C\C=C\CCCCCCCCC(=O)OC(CC(O)=O)C[N+](C)(C)C |
| ACar 18:3 | GCEIHHLHHYUVPL-OBWVEWQSNA-O | CC\C=C\C=C\C=C\CCCCCCCCCC(=O)OC(CC(O)=O)C[N+](C)(C)C |
| ACar 20:1 | PVOQPLSKYNZHOT-OUKQBFOZNA-O | CCCCCCCC\C=C\CCCCCCCCCC(=O)OC(CC(O)=O)C[N+](C)(C)C |
| ACar 20:3 | DBCKADUSYJAPII-LJURNQLPNA-O | CCCCCCCC\C=C/C\C=C/C\C=C/CCCC(=O)OC(CC(O)=O)C[N+](C)(C)C |
| ACar 20:4 | RBFQHRALHSUPIA-WFYBHXQRNA-O | CCCCC\C=C\C\C=C\C\C=C\C\C=C\CCCC(=O)OC(CC(O)=O)C[N+](C)(C)C |
| ACar 26:0 | KOCKWDDTAHPJSX-UHFFFAOYNA-O | CCCCCCCCCCCCCCCCCCCCCCCCCC(=O)OC(CC(O)=O)C[N+](C)(C)C |
| ACar 26:1 | HDCKEMMZMZBAHM-VHEBQXMUNA-O | CCCCCCCCCCCCCC\C=C\CCCCCCCCCC(=O)OC(CC(O)=O)C[N+](C)(C)C |

**Supplementary Table 2. LASSO, ENET, WQS models to investigate the associations of** **plasma acylcarnitines with diabetic retinopathy.**

| **Plasma**  **ACars** | **LASSO** |  | **ENET** |  | **WQS** |
| --- | --- | --- | --- | --- | --- |
|  | **Coefficients*^a^*** |  | **Coefficients*^a^*** |  | **Weights*^b^*** |
| **ACar 8:0** | **-0.115** |  | **-0.115** |  | **1** |
| ACar 8:1 | - |  | - |  | 0 |
| ACar 10:1 | - |  | - |  | 0 |
| ACar 11:1 | - |  | - |  | 6e-07 |
| ACar 12:0 | **-** |  | - |  | 1e-07 |
| ACar 12:1 | - |  | - |  | 0 |
| ACar 13:0 | - |  | **-** |  | 0 |
| ACar 14:1 | - |  | - |  | 0 |
| ACar 16:0 | - |  | - |  | 0 |
| ACar 16:1 | - |  | - |  | 0 |
| ACar 16:2 | **-** |  | - |  | 0 |
| ACar 18:0 | - |  | - |  | 0 |
| ACar 18:1 | - |  | - |  | 0 |
| ACar 18:2 | - |  | **-** |  | 0 |
| ACar 18:3 | - |  | - |  | 0 |
| ACar 20:1 | - |  | - |  | 0 |
| ACar 20:3 | **-** |  | - |  | 0 |
| ACar 20:4 | - |  | - |  | 0 |
| ACar 26:0 | - |  | - |  | 0 |
| ACar 26:0 | - |  | - |  | 0 |

***Notes:*** *^a^ Coefficients were obtained from the models without penalization covariates;*

*^b^ weights were obtained from the models with adjusted covariates, include age, sex, BMI, smoking habits, alcohol consumption, education, duration of diabetes, TG, FPG, SBP, and Center; Bold values refer to selected variables; In the WQS regression models, the cutoff of the weights was 0.05; weights may not sum to 1 because of rounding.*

***Abbreviations:*** *LASSO: Least absolute shrinkage and selection operator; ENET: Elastic net; WQS: Weighted quantile sum, ACar: Acylcarnitine.*

**Supplementary Table 3. The relation between Acar8:0 and clinical characteristics.**

| **Variables** | **Acar 8:0** | |
| --- | --- | --- |
|  | **Correlation coefficients** | **P values** |
| **Age** | -0.086 | 0.31 |
| **Gender** | -0.136 | 0.11 |
| **BMI** | -0.013 | 0.88 |
| **HbA1c** | 0.098 | 0.25 |
| **SBP** | -0.142 | 0.10 |
| **Center** | 0.033 | 0.70 |
| **FPG** | 0.123 | 0.15 |
| **TG** | 0.176 | 0.04 |
| **Smoking habits** | 0.077 | 0.37 |
| **Alcohol consumption** | 0.023 | 0.79 |
| **Education** | 0.038 | 0.66 |
| **Duration of diabetes** | -0.153 | 0.07 |

***Notes:*** *The spearman correlation coefficients were used to characterize the correlation between Acar8:0 and clinical characteristics.*

***Abbreviations:*** *ACar: acylcarnitine.*

**Supplementary Table 4. Sensitivity analyses of the association between Acar8:0 and the risk of DR.**

| **Analyses** | **ACar 8:0 (μmol/L)** | | | ***P*** *_for trend_* |
| --- | --- | --- | --- | --- |
|  | **T1 (n=46)** | **T2 (n=46)** | **T3 (n=46)** |  |
| **No processing of missing values** |  |  |  |  |
| Model 1 | 1∙00(ref.) | 0.24(0.10,0.59) | 0.14(0.06,0.35) | <0∙001 |
| Model 2 ^a^ | 1∙00(ref.) | 0.23(0.08,0.62) | 0.11(0.04,0.36) | <0∙001 |
| Model 3 ^b^ | 1∙00(ref.) | 0.29(0.09,0.89) | 0.11(0.03,0.38) | 0.001 |
| **Filling in missing values** |  |  |  |  |
| **Adjusting for variables associated with dietary variations** | | | | |
| Model 1 | 1∙00(ref.) | 0.24(0.10,0.59) | 0.14(0.06,0.35) | <0∙001 |
| Model 2 ^c^ | 1∙00(ref.) | 0.30(0.10,0.94) | 0.11(0.03,0.38) | 0∙001 |
| **Adjusting for ever Insulin therapy** |  |  |  |  |
| Model 3 ^d^ | 1∙00(ref.) | 0.22(0.06,0.77) | 0.07(0.02,0.32) | 0.001 |
| ***Notes:*** *Model 1: unadjusted for potential confounders.*  *^a^ adjusted for age, sex, BMI, smoking habits, alcohol consumption, education, TG, FPG, SBP, and Center.*  *^b^ adjusted for age, sex, BMI, smoking habits, alcohol consumption, education, duration of diabetes, TG, FPG, SBP, and Center.*  *^c^ adjusted for age, sex, BMI, smoking habits, alcohol consumption, education, duration of diabetes, TG, FPG, SBP, Center, and usual consumption of oils and salt (high, middle, low).*  *^d^ adjusted for age, sex, BMI, smoking habits, alcohol consumption, education, duration of diabetes, TG, FPG, SBP, Center, and insulin therapy.*  ***Abbreviations:*** *DR: type 2 diabetic patients with diabetic retinopathy; T1, T2, T3: the 1^st^, 2^nd^ and 3^rd^ tertile of ACar 8:0, respectively; OR: odds ratio; CI: confidence interval, ACar: Acylcarnitine, P _for trend_: P values for testing the linear trend between DR and ACar 8:0.* | | | | |

**Supplementary Table 5. AUC and *P* values of DeLong's test of the prediction model of the ACar 8:0.**

|  | AUC (95%CI) | Sensitivity | Specificity | PPV | NPV | *P* values of Delong's test |
| --- | --- | --- | --- | --- | --- | --- |
| Ref. | 0.67 (0.58,0.76) | 68.12 | 60.87 | 63.51 | 65.63 | - |
| ACar 8:0 | 0.74 (0.66,0.82) | 59.42 | 81.16 | 75.93 | 66.67 | 0.248 |
| Ref.+ACar 8:0 | 0.77 (0.70,0.85) | 82.61 | 65.22 | 70.37 | 78.95 | 0.011 |

***Notes:*** *The reference model included smoking habits, alcohol consumption, education, SBP, TG, FPG and duration of diabetes; the P values of Delong’s test were obtained by comparing with the reference model.*

***Abbreviations:*** *AUC: areas under the ROC curves; DR: type 2 diabetic patients with diabetic retinopathy, ACar: acylcarnitine, PPV: positive predictive value, NPV: negative predictive value.*

**References**

1. Wilkinson CP, Ferris FL, 3rd, Klein RE, Lee PP, Agardh CD, Davis M, et al. Proposed International Clinical Diabetic Retinopathy and Diabetic Macular Edema Disease Severity Scales. *Ophthalmology* (2003) 110(9):1677-82. doi: 10.1016/s0161-6420(03)00475-5.

2. Ophthalmoscopy D, Levels E. International Clinical Diabetic Retinopathy Disease Severity Scale Detailed Table (2002).

3. ROSENBAUM PR, RUBIN DB. The Central Role of the Propensity Score in Observational Studies for Causal Effects. *Biometrika* (1983) 70(1):41-55. doi: 10.1093/biomet/70.1.41.
